# "The Hospital" Nursing Mirror

**Published:** 1901-01-12

**Authors:** 


### "The Hospital" Nursing Mirror.

Being the Nursing Section of "The Hospital."

[Contributions for this Section of "The Hospital" should be addressed to the Editor, "The Hospital" Nursing Mirror, 28 & 29 Southampton Street, Strand, London, W.C.]

### Notes on News from the Nursing World.

### THE REWARD OF HEROISM.

Miss Eulalia Bourguignon, one of the nurses who has just received the valued decoration of the Royal Red Cross from the Queen, was the only trained nurse in Tien-tsin during the first awful days of the siege. She herself was wounded by a shell, in spite of which she continued nursing without cessation. This is no doubt one of the reasons for which she was awarded the distinction, and it will be admitted that she richly deserved it. Miss Bourguignon is a member of the Royal National Pension for Nurses.

### MEDALS FOR PLAGUE NURSES.

The new year at Belvidere Fever Hospital, Glasgow was ushered in by a band of nurses serenading the matron and afterwards the patients with "A guid New Year to ane an' a'." On New Year's Day the nurses partook of an excellent dinner, after which the annual visit of the Lord Provost and committee took place. The hall was tastefully decorated, and when filled with the nurses in their pretty uniforms had a charming effect. After addresses given by Lord Provost Chisholm and several members of the committee, medals were given to those who had nursed the plague patients. The medals were presented by the Lord Provost and pinned on the recipients by Mrs. Sinclair, matron. It was gratifying to see how the whole staff rejoiced in the honour paid to their fellow nurses; but the climax was reached when the Lord Provost, in a few happy sentences, presented Mrs. Sinclair with a similar medal. The cheering was loud and long, and showed clearly the esteem in which the matron is held by her nursing staff. After a few remarks from Dr. Brownlee, medical superintendent, who was also greeted with applause, the proceedings closed, everyone feeling that the New Year had commenced most auspiciously in Belvidere Hospital. The medals, which are the gift of the City of Glasgow Corporation, are of gold in the form of a cross with the City of Glasgow coat-of-arms engraved on one side and on the other the nurse's name, the date, and the word "Plague."

### LORD ROBERTS AND THE ALEXANDRA NURSE.

Although Lord Roberts had only a limited time in Maritzburg, he succeeded in visiting the Soldiers' Institute. Unfortunately, the funeral of an officer in camp prevented some of the men from being present, but those who were fortunate enough to see the Field-Marshal were much gratified by the keen interest he showed in everything concerning them. He went all over the Institute; shook hands with several ladies who had been especially kind to the soldiers in many ways; wrote his name in the Birthday Book of the Misses Walker, to whom the Institute is much indebted, and also some kindly words in the visitors' book; addressed a few remarks to the senior non-commissioned officer and chatted for some little time with the Alexandra nurse, as representing the women and children of the regiments, enquiring in a most kindly manner about them. As may be imagined, when the nurse went her rounds the next morning, and was able to tell her patients of the nice way in which Lord Roberts had spoken about them, her communication was received with much pleasure. One woman, the wife of a sergeant, now the mother of four little ones, was especially pleased to hear anything respecting the Commander-in-Chief, because some years before, when her first baby had been born in India, Lady Roberts had not only visited her, but had given her some good advice as to the future up-bringing of the little stranger—advice which had not only proved useful, but had always been gratefully remembered by the recipient.

### AN INGENIOUS NEW YEAR'S CARD.

An Army Nursing Reserve sister, who is stationed at Pretoria, finding a New Year's card an unobtainable article, decided to manufacture some, which she did in the following manner. A pair of soldier's "unmentionables," which, by the way, had been through the siege of Ladysmith, were washed and cut up into neat patches about five inches square. The edges were frayed out, and the pieces smoothly ironed. Then, in red and black ink, a geometrical design was sketched as a border, and in the centre was inscribed: "'A Khaki Patch.' To convey my Good Wishes for the New Year and the New Century.
*To* . *From*
Boer War: Pretoria, 1900-1901." These unique New Year's cards are much prized by their recipients.

### SLAVERY AT SYDNEY.

The hours of labour of the nurses of the Sydney Hospital have been brought under the notice of the Legislative Assembly. According to the *Sydney Daily Telegraph* of November 28th, Mr. Holman informed the Assembly that the nurses were kept at work for no less than eighty hours per week, with only one full holiday of twenty-four hours in the year. He contended that it was impossible for them to perform their very delicate duties efficiently under "such inhuman conditions." The Premier said that "he had already heard rumours with regard to the way in which the nurses of the Sydney Hospital were treated, and he had given instructions that these ladies were not to be worked like slaves." When it becomes necessary for the Legislature to intervene for the protection of the nurses of a hospital, the time for sweeping changes in the institution has clearly arrived.

### MATERNITY NURSE v. MIDWIFE.

The majority of members of the Rugby District Nursing Association think that the poor women of the town prefer the services of a certificated midwife, who is supposed to be qualified to take the case alone, to a trained maternity nurse who works under a doctor, and they have accordingly decided to engage the former. But there was a dissenting minority. The Rector of Rugby, who said that "if they were going to give it out that they were about to employ a midwife without emphasising that the proper thing was to employ a doctor he should be very sorry for it," yet voted for the midwife's appointment, delivered himself into the hands of Dr. Henderson, who contended that a doctor should always be present the first part of the time. No doubt, it is better that the poor women of Rugby should be attended by a certificated midwife than by the untrained persons who have been in the habit of rendering such assistance as they could. But it is a pity that the Association should have been influenced, even, as we gather, by the fear of extra expense, not to appoint a maternity nurse. This was the course favoured by several of the medical men in the town, and we agree with Dr. Henderson that an institution like the Rugby Nursing Association ought to be able to pay the fee of the doctor.

### LORD ROBERTS ON THE "AVOCA."

THE patients who came home on the hospital ship *Avoca*, upon the occasion of her last voyage to England, were honoured before leaving Durban with a visit from Lord Roberts. The day previous to his sailing in the *Canada* he went all over the *Avoca*, interviewing each patient, asking the men their names, regiments, the nature of their wounds or diseases, and spending some little time with Sergeant Nurse V. C., who had been with Lord Roberts' son when the latter was wounded at Colenso. Before leaving the Field-Marshal expressed himself thoroughly satisfied with all the arrangements which had been made for the comfort of the men. There were no deaths upon the return voyage, though five of the 269 men were very ill, and it was scarcely thought that they would reach England alive. All those who were able to partake of seasonable fare on Christmas Day were given eggs and cocoa, jam and marmalade, in addition to porridge for breakfast; whilst those who were not well enough to eat plum pudding had fresh fish and fruit. Great care had to be exercised to prevent the good things from being given to the wrong patients. A glass of port was allowed to each man after dinner, and a concert was held in the evening. Besides the 291 patients, there were 23 sick officers, including two invalid sisters of the A.N.S.R.

### LADY AUDREY BULLER ON MATERNITY NURSING.

AT the annual meeting of the Crediton Sick Nurse Association Lady Audrey Buller advocated the employment of a maternity nurse, and said she was very anxious about the subject as the result of her experience at Aldershot. At Crediton there was no certificated maternity nurse, and it was most sad and painful, she thought, that a woman who could not have a doctor to consult should be in the hands of people who were not fit to look after her. She offered to come from Aldershot later on to a special meeting, and she advised that a portion of the endowment fund of the Association should be used as an experiment. The suggestion was made, not with the idea of superseding, but of supplementing, the services of the present nurse. There is every reason to believe that a satisfactory response to the proposal will be made by the Crediton people, if only to show their high appreciation of the work done by General Buller in South Africa.

### UNTRAINED MEN TEACHING GIRLS NURSING.

UNDER the heading of "The Degradation of Nursing" we publish in another column a letter from an ex-nurse drawing attention to the extraordinary course pursued by the London School Board. She affirms that "unqualified men are allowed by the London School Board to teach home nursing to girls and women," and in corroboration of her statement she encloses the Evening Schools Agenda of the London School Board, dated December 17th, 1900. There are two entries which fully establish her case. One is to the effect that the home-nursing class for females at Thornhill Road, Finsbury, is taught by a man who holds "the St. John's Ambulance Association certificate in home nursing," but has not received recognised training as a nurse. In the other case a man has been employed at three schools in South London to take female classes in ambulance and home nursing; and, though no mention is made of any training, according to this one's letter, he has worked to "the full knowledge of members of the Board, Board inspectors, managers, and Government inspectors.' This condition of things is not to be continued during the present year; but the fact that it has been going on for some time shows, in a conclusive manner, the incapacity of the London School Board to deal at all with the question of nursing. In the event of the evening classes being resumed, we trust that the ratepayers who are interested in nursing will insist upon any instruction in home nursing given to girls being placed in the hands of trained female nurses. They are, obviously, the proper persons to afford it. For the rest, our correspondent, who declares that the action of the London School Board is a degradation to her profession and an insult to her sex, does but echo the indignation which will be felt among nurses generally when they learn how strangely an important public body has misinterpreted one of its serious obligations.

### SHAM NURSES.

IT is satisfactory that one, at any rate, of the numerous persons who masquerade as nurses, has had her career cut short for a season. She had represented herself as a nurse at the Women's Hospital, Sparkhill, Birmingham, and attired in professional costume, had obtained a loan of thirty shillings at the Home for Little Sisters of the Poor, Harborne, on the faith of her statement. As the money was not returned at once, in accordance with her promise, inquiries were set on foot, and it was found that she had never been a nurse at the Sparkhill Hospital. When she was charged with fraud at the Birmingham Police Court, she adroitly pleaded that the money was only a loan. But the Bench very properly convicted her of obtaining the loan under false pretences and sent her to prison for a couple of months. The sentence standing alone would seem severe, but evidence was given at the police-court which proved that she had been getting money and food in many quarters on similar grounds. It is curious that the people at the Home for Little Sisters of the Poor did not test the truth of the woman's statements before giving her money. If the matron of the hospital at Sparkhill had been applied to, she could at once have disposed of them. It may not be possible to put an end to the malpractices of sham nurses, who are often exceedingly cunning. But when they ask for help on the ground of a connection with a particular institution, near or far away, it is easy to ascertain whether their claims are valid. Another impostor is the subject of a warning by the steward of St. Thomas's Hospital. Mr. Sydney Phillips says that a young woman giving the name of Norah Campbell is representing herself as a sister or staff nurse at St. Thomas's, and is obtaining money from people under various pretences. No such person has ever been a nurse at that hospital.

### MISS TWINING ON WORK AND DIET.

We publish in another part of the paper a letter from Miss Louisa Twining, whose earnest efforts to promote the improvement of the conditions of nursing in workhouse infirmaries entitle her to a respectful hearing on the work and diet of nurses and probationers. There is a good deal in her communication with which we agree, but so far as hospitals are concerned we certainly adhere to the conviction that the work of the nurses has been greatly diminished, and the diet greatly improved, during the twenty years of Miss Twining's association with nurses. There is much force in our correspondent's remarks on the need of appointing women as inspectors of workhouses, though, as Miss Twining admits, that is another subject.

### PORTSMOUTH CORPORATION AND TRAMWAY PASSES.

The tramways at Portsmouth have just been acquired by the Corporation, who have announced that "no free passes will be allowed." It is naturally feared that this applies to the nurses of the Royal Hospital, who for some years have enjoyed the privilege of being allowed to travel free of charge on all the trams in the Portsmouth district. As the Royal Hospital is situated in the most crowded part of the town, a mile away from the sea or the country, the concession has been greatly appreciated, and its withdrawal will be correspondingly felt by a class of workers who in that case must spend a considerable proportion of their not too large salaries on their daily outing.

### NORTH DEVON INFIRMARY, BARNSTAPLE.

We continue to receive complaints about the state of affairs at the North Devon Infirmary. We cannot but feel that unless the authorities of this infirmary adopt a more conciliatory tone towards their subordinates, they will find it extremely difficult to replace the probationers whom they have somewhat summarily dismissed.

### A CRY FROM WHITBY.

The correspondent who, in the interests of patients and nurses, originally complained of the site of the new permanent cottage hospital at Whitby in sending us a photograph showing the building, writes:—

There is a lovely view from the house, as the secretary says, at high tide, and at low tide the mud banks often take beautiful colours; but are they healthy? The smell from them in hot weather is very bad. At low tide there is only a narrow stream in the middle of the river bed and a great expanse of mud banks. My authority for the water in the cellars is a woman who lived in the house as servant for many years. She says that there was sea water in the cellars at every tide, often a great deal of it. The secretary says that the old part of the bank is to be pulled down. I believe the whole building is very old—nearly 200 years old in fact. I know that a great many people in Whitby think this a very bad place for the cottage hospital, and much wish to have the question reconsidered. A larger cottage hospital is very much needed in Whitby, and the present little one has proved most useful; so it would be very sad if the usefulness of the larger one were to be imperilled by its being in a bad situation.

With regard to the photograph our correspondent says:—

It was taken at about half-tide. You will see three drain-holes just under the window of what is to be the principal ward. They discharge their contents on to the mudbank at low water, and at a very high tide the river gets into them.

This last statement, however, does not necessarily strengthen the case against the site, as the drain holes are probably only surface drains.

### VISITING NURSES FOR THE MIDDLE CLASSES.

In reference to an inquiry by a correspondent last week, we learn that the Association of Visiting Nurses for the Middle Classes is being proceeded with. A printed circular enclosed by our correspondent describes the scheme as "particularly adapted to those living in flats and chambers," and mentions the fees as ranging from 2s. 6d., for an ordinary simple medical case for a visit not exceeding two hours, to 10s. 6d., and 15s. 6d. for operation and accouchement cases. But we should like to know of whom the Association is, or is to be, composed. To obtain confidence and success there should be a strong committee. A single name on a circular is not sufficient.

### OPENING OF THE NURSES' HOME AT TAUNTON.

Last week the new Nurses' Home at Taunton was opened in the presence of a large gathering. As already announced in our columns, the Home is the gift of Mr. George Saunders, of Bishop's Lydeard, who has always taken a great interest in the nursing movement. At the conclusion of the proceedings the company inspected the building, in the hall of which are hung portraits of Miss Ethel Fisher and Nurse Sage, whose deaths occurred last year.

### CHILDREN'S ENTERTAINMENT.

On Friday the children at Paddington Green Children's Hospital had their entertainment, consisting of conjuring with ventriloquism and an excellent magic lantern display, both of which the little patients keenly enjoyed. Flags and evergreens were used in decorating the wards, and in one was an enormous cracker, 14 feet long, the gift of Mr. Thomas Gibbs. When opened it was found to contain about 1,400 smaller crackers. The decorations were arranged and carried out by the matron and nurses.

### SOUTHGATE VILLAGE NURSE FUND.

An interesting feature of the annual report of the Southgate Village Nurse Fund is that the house-to-house collection amongst the poor yielded a sum of £6. The number of visits paid in 1900 was 4,014, as against 1,695 in 1896, the year the charity was started; a fact which shows how increasingly it is appreciated. But the fund has not yet been put on a permanent basis, part of the house rent being paid for privately. It is desired to buy a small house for the use of the nurse, and, as it is stated that three or four persons have promised their help in the event of a suitable one being found, the wish and the fulfilment should not be far apart. The nurse has been asked to visit the inmates of the almshouses at Palmer's Green, and the Skinners' Company, to whom they belong, have granted a liberal donation.

### SHORT ITEMS.

The annual general meeting of the Midwives' Institute and Trained Nurses' Club will be held on the evening of Friday, the 25th inst., at 7 o'clock.—In the list of Queen's nurses last week, the name of Miss Annie Button was wrongly given in the copy supplied to us as "Bulton."—In Mrs. Goslett's examination in hygiene, held at Bloomsbury on December 10th, the results of which have just been received, Miss Grace Vaughan secured the highest number of marks. Miss Vaughan is engaged in nursing in the Fulham and Hammersmith District.—Among the numerous victims in a disastrous fire near Rochester, New York, on Tuesday, which resulted in the destruction of the west wing of the Hubbell Park Orphan Asylum, was Miss Giles, a nurse, who fell a depth of three storeys to the ground, and was instantly killed.—The Queen has sent a present of twenty pheasants for the patients of the Cancer Hospital, Fulham Road.

### Lectures to Midwifery Nurses.

By ROBERT JARDINE, M.D., &c., Physician to the Glasgow Maternity Hospital. Examiner in Midwifery to the University of Glasgow, &c.

### SOME OF THE BLADDER COMPLICATIONS OF LABOUR.

FREQUENCY of micturition is very common in labour, especially during the first stage; the patient will have an almost constant desire to pass urine. In other cases again you will find incontinence present. With each pain some urine will dribble away. Neither of these conditions are of any consequence except for the discomfort. They do not delay labour, but when there is incontinence the patient is almost sure to inform you that the "waters have broken."

Retention of urine occasionally occurs. It is a more serious condition as it retards labour in both the first and second stages. In the first stage the distended bladder tends to prevent the presenting part entering the brim, and thus interferes with dilatation of the os. In the second stage it not only blocks the way to a certain extent, but it also interferes with the action of the secondary powers. The woman cannot bear down properly.

The patient before us is a primipara who has been in labour for some hours. The os is fully dilated, membrane ruptured, and the head low in the pelvis. She is having good pains, but there is very little progress being made. When you look at the abdomen you at once perceive that it is not the usual shape. Just above the symphysis you notice a swelling, and then a depression or sulcus running somewhat obliquely across, a little below the umbilicus. Above this we see the ordinary uterine enlargement. To the feel the lower swelling seems full of fluid. It does not contract like the upper part when a pain is on. It becomes somewhat tenser during a pain, but that is evidently due to pressure exerted by the contracting uterus behind coming somewhat forward. It is quite evident that we have here to deal with a moderately distended bladder. The patient says she has not passed urine for many hours.

There is a condition of the uterus which gives you the same appearance of two swellings with a deep groove between them—viz. when Bandl's ring has become very apparent. This condition is, I believe, what is sometimes described as "an hour-glass contraction of the uterus." It is a very serious condition, as the lower uterine segment is then stretched to its utmost, and rupture of the uterus is imminent if it has not already occurred. We shall not discuss this further, but return to the consideration of the distended bladder.

*Treatment.*—We must pass a catheter and draw off the urine. When this is done I have no doubt the labour will soon be finished. The catheter may be passed with the patient lying on her left side or on her back with her legs drawn up. As the head is low down there will be some difficulty in getting the instrument in. Sometimes a flexible instrument has to be used, but gum elastic catheters are very difficult to sterilise, so I prefer a long silver one. The ordinary female catheter is rather short. A glass instrument is very good during the puerperium or at other times, but not during labour, as it might be broken in unskilful hands. Whatever instrument is used it should have a solid point beyond the eye, or else have the opening at the end. If the part beyond the eye is hollow it cannot be properly cleaned and septic material may easily be carried into the bladder. The catheter we are to use is a long silver one only slightly curved. It has just been boiled and placed in lysol solution. The lysol lubricates it. The patient is now placed on her back in the lithotomy position. The external genitals are thoroughly washed with lysol solution and the meatus carefully cleansed. On introducing the catheter it only enters about an inch when it is stopped. Force must not be applied. It is the head pressing upon the urethra which blocks the way. You will observe we are doing this in the interval and not during a pain. To get the instrument to pass the head must be pushed up by two fingers introduced into the vagina. The fingers must, of course, be thoroughly clean. When the head is pushed up you see the catheter slips in easily enough. When the catheter is withdrawn your finger should be placed over the outer end of it, as this will prevent the urine in it from running on to the patient or bed. Some of you may say, why did you not pass the instrument by touch under the bedclothes, and not expose the patient? It is easy enough to do it in the dark and under the clothes, but it is practically impossible to do it aseptically in that way. Your eyes will never do any harm, but your fingers feeling for the meatus and touching different parts may very easily contaminate the catheter, with the result that septic material will be carried into the bladder and cystitis or inflammation set up. During the puerperium you should certainly always see what you are doing when passing the catheter, and make sure that all the lochial discharge is carefully wiped away from the vulva and meatus. Lochial discharge, which has been exposed to the air, is sure to be swarming with various forms of micro-organisms, and if any of it is introduced into the bladder, cystitis is almost sure to arise. I have never yet found a patient object to the exposure, and if she should, a short explanation of why it should be done by sight and not by touch would no doubt remove her objections. I can see no more reasons for passing the catheter under the bedclothes than for applying the forceps in the same way. They are both operations which require to be done with the strictest aseptic precautions, and these can only be carried out by sight.

There is another bladder complication which sometimes gives a good deal of bother, viz., a cystocele or prolapse of a portion of the bladder in front of the presenting part. The anterior vaginal wall protrudes at the vulva. You may mistake this for a prolapsed and œdematous anterior lip of the cervix, but a careful examination will reveal that it is the anterior vaginal wall and not the cervix.

*Treatment*: Empty the bladder, if there is any urine in it and then push the vaginal wall up in the intervals between the pains, and support it during a pain until you get it above the head.

### Appointments.

WISBECH UNION INFIRMARY.—Miss Jenny Mabel Watts has been appointed superintendent nurse. She was trained at the Royal Hants County Hospital, Winchester, and has since been home sister at York County Hospital, and home sister at the Royal Hants County Hospital.

IPSWICH UNION INFIRMARY.—Miss E. E. Ingram has been appointed superintendent nurse. She was trained for three years at the Devon and Exeter Hospital, where she was subsequently charge nurse. She has also been charge nurse of wards for seven years at Mill Road Infirmary, Liverpool. Miss Ingram holds the L.O.S. certificate.

BRISTOL ROYAL INFIRMARY.—Miss Mary Stanford has been appointed ward sister. She was trained at the Brompton Hospital and St. Thomas's Hospital, and has since been staff nurse at University College Hospital.

STOCKTON AND THORNABY HOSPITAL.—Miss Olive K. Heath has been appointed matron. She was trained at the General Hospital, Dudley, and the East Dulwich Infirmary. Subsequently, she has been charge nurse at the Surgical Hospital, Barnsley; district nurse at Dodsworth, Barnsley; head nurse at Greenwich Infirmary, charge nurse at Stockton and Thornaby Hospital, and matron for three and a-half years of Keighley Infirmary.

### The First Nurses at Waterval Onder, Transvaal.

By ONE OF THEM.

Almost the last place of any importance we stopped at before coming here was Pretoria, which was reached late on a Saturday evening, and we slept on the train till Monday. As we had been three days and three nights on the journey, it may be imagined that we were train-sick, especially as in hospital trains there are no seats. Fortunately as we had taken our folding chairs, we were not so badly off.

On Monday morning we had received no further orders, but as the train was to be shunted into a siding, the Major thought we had better leave our goods on the platform, so we finally settled ourselves on our traps, "waiting to be called for," looking tragic in our bonnets, which for this important occasion we had deemed necessary. The Major told us that the P.M.O. would probably send orders about 1 P.M. so as we were desperately hungry we went off to the Grand Hotel, leaving an orderly on guard. Luncheon did much towards reviving us and we came back to the station about 2 P.M. to find the Major stamping up and down because he had got a good "rowing" on our account. We were told to get on to the train and stay there.

### Travelling in a Box Truck.

During the afternoon the P.M.O.'s secretary arrived with the order that we were to proceed to Waterval Onder, but the difficulty was how to send us. It was left to us to choose whether we would go on at once in a box-truck attached to a store train, which would take from three days to a week, and run the risk of being attacked by the Boers who were playing about, or wait perhaps a fortnight for Princess Christian's hospital train, and in the meantime be attached to a hospital in Pretoria. We decided on the former plan, the novelty of the box-truck appealing to us, and next morning we were waiting on the line with our boxes all around, like "'Arriet going to her first place."

The train stopped, we climbed up, our boxes being set round us again. What should we have done without our chairs? There was absolutely nothing in the truck. Still, we had a cover, the other few passengers being in open trucks. One of them, a chaplain, took an interest in us, and came every time the train stopped to see if he could do anything. How that train did "waggle"! Our boxes constantly made for the door, and we after them at the risk of being pitched out head first.

### Tommies make Tea.

At the stations we were always surrounded by soldiers anxious to know where we were going. At one wild-looking place we noticed about eight men busy over a fire in the open, on which they had a big dicksie. We wondered what it contained, their excitement was so great. They blew up the fire, peeped inside, and altogether looked very anxious. Finally, we saw one of them walking carefully towards the train, carrying a dish of some sort in each hand. He came up to us and said, "We thought maybe, you two sisters would be glad of some tea. Sorry we have no cups." The tea was in two small basins set on plates, and all beautifully polished. It was a godsend to us! The man returned at the right time for the dishes; and hoped that he had not "'ad too 'eavy a 'and" with the sugar.

### Our Arrival.

At Middleburg we were to stay the night. What was to be done? Must we sit in that truck all night? Our good friend the chaplain asked permission to escort us to the town. He took us to the Sisters there, who entertained us to dinner and went with us to interview the R.S.O. as to when the train would leave next day Finding it would not do so before 9 A.M. they put us up for the night, and gave us a good "send off" next morning.

We are the first two nurses to go so far into the Transvaal. A doctor along the line said he thought it was not very fair to send us, as the Boers were all around, and that, only the night before, a train had been attacked, five Coldstream Guards killed and twenty-one wounded. We saw the graves as we passed. We arrived at 8 P.M. A soldier offered to escort us to the hospital. We asked for the S.M.O., and he came, looking very flustered. We were not expected. The wire from P.M.O. Pretoria had never been sent. The S.M.O. ushered us into a queer little shanty, where he, with two guests, had just finished dinner. He managed to do the right thing, and ordered dinner and champagne for us. Then began a discussion as to what was to be done with us. There were only half a dozen houses in the place, and they were absolutely empty. At last one of them thought of the hotel, and went to interview the proprietor, who, being a Frenchman, said it was "Impossible." However, the Provost Marshal said it must be, and he had to rig us up a room.

### The Hospital.

Next day an empty house was chosen, and a gang of natives set to clean it. All sorts of things began to arrive, brought by all sorts of men. The R.S.O. himself arrived with a bit of a rug for the sitting-room. The furniture consists of two beds, four chairs, two toilet sets, two washstands, a table, a kettle, a saucepan, and a frying-pan. We have three rooms, and a nice cool verandah with passion flowers creeping over it. In front are big gum trees, which we glory in; they make the house so cool. Our garden contains lettuces, carrots, beans, beetroot, &c.

Our domestic is one-footed, and his name is Sugar. He is a very good boy, and quite the joy of our lives. All the English he can manage amounts to "Yas, Missus." Waterval Onder, as its name implies, lies, as it were, under the water. Waterval Bovan is the station; and there is an immense waterfall. It is in a low marshy valley, with miles and miles of mountains in every direction. The hospital, which consists of two buildings, has been here for a good while, and has been managed by the Dutch. It is now one of the Imperial Railway hospitals. It is worked neither on military nor on civil lines—just any way. The Dutch nurses leave this week. It is very weird to be cut off from everyone. We are the only English women in the place. We cannot go far away, as the outposts are quite near and turn us back. The climate is bad, and Blackwater fever is common. Frogs, lizards, locusts, and beetles abound. This hot, damp, steamy ground is just the place for loathsome creatures. A snake was found at our door the other day.

### How we Live.

We live in a hand-to-mouth sort of way, just picking up what we can. Oh, for a decent English meal! Here it is tinned butter, tinned milk, tinned jam, tinned everything, boiled rice, trek ox, and bread, sadly underdone. My colleague laughs at me and says I am too fastidious, and that I should not pull every ant's leg out of the butter, but my friends may be gratified to know that I have not yet come to eating ham with my fingers.

I do not think there is much prospect of getting home yet awhile. I cannot say I am in a hurry. Still, I wish the war would really end.

### With Lord Roberts from the Cape.

BY A CORRESPONDENT.

### A CHAT WITH TWO NURSING SISTERS.

THE nursing sisters on board the ss. *Canada*, in which Lord Roberts made the voyage home, were Sister-Superintendent Laurence (who was Superintendent of the Princess Christian Hospital, Pine Town Bridge, Natal), and Sisters Fisher, Balfour, and Coutts. Sisters Skillman and Smith, who nursed Miss Roberts through her attack of enteric in Pretoria, also travelled home with her; and there were three Roman Catholic Sisters on board who had been nursing in Bloemfontein.

From Sisters Laurence and Balfour I have just heard some interesting details of the voyage, and of course one of the first questions I asked was, "Did you see much of Lord Roberts?"

### DISTINGUISHED PASSENGERS.

"We saw him constantly; both he and Lady Roberts took the greatest interest in the sick men. Indeed no one could have been more thoughtful and kind in making daily inquiries after the sick. More than once they went through the hospital, asking the men how they were, and what regiment they belonged to. He took a great interest in us, too, and wanted to know what we were going to do."

There were on board, besides Lord and Lady Roberts and their two daughters and all the staff, Generals Kelly Kenny, Ian Hamilton, and Marshall, and Mrs. Knox (wife of General Knox), was also among the passengers. About twelve officers were invalided home, and the "Times" correspondent, who was very badly wounded in the foot, was carried on deck every day.

### "BOBS' CAKE."

"On Christmas Day," said Sister Laurence, "Lord Roberts went through the hospital and shook hands with every patient, and he sent a large sugared cake down for the men who were well enough to enjoy it, but I don't think many of them ate their portion! There were many demands for envelopes in which to "take Bobs' cake home." No one could have been more thoughtful and kind than Lord and Lady Roberts, and many of the beautiful baskets of flowers which came on board for Lady Roberts at the various ports we called at found their way to the hospital. The men especially treasured a Union Jack, made of red geraniums and blue and white violets, which came on board at Madeira."

### HOW THE WORK WAS DONE.

"Had you hard work during the voyage?" I asked.

"No, the work was not heavy, but it was very anxious sort of work," Sister Balfour replied. "We had two deaths during the voyage, and five other very bad stretcher cases, whom we saw safely off for Netley on arrival—three enterics and two pneumonias."

"The duty was equally divided?"

"Yes; and we took turns at night duty, so that it came to each every fourth night. This did not mean necessarily staying up all night, unless there was special cause, but staying on till midnight and then being called again to make a round about 4 a.m."

### SOME SPECIMENS OF WEATHER.

"Was the passage good?"

"The weather was very fairly calm all the way," said Sister Laurence, "but we came in for rather a bad roll in the Bay. The ship's boy remarked, 'Fresh to moderate gale with confused sea'! It was bad for our poor men with enteric, and even the two sisters who were able to keep about did not enjoy having to sponge men in lower berths when every roll nearly upset the water either on to the floor or over the patient."

"Was much illness developed on board?" I inquired.

"Four men developed enteric," Sister Laurence said, "after coming on board, and two of them were very ill indeed with pneumonia complications. We had three of them in swinging cots on the upper deck all through the very hot weather in the tropics, as the wind was following us and it seemed impossible to keep their temperatures down at all in the somewhat airless hospital; but there was always a good supply of ice."

The food, Sister Laurence added, was excellent; the men were delighted with the variety supplied by the Company (the *Canada* belongs to the Dominion Line), and as there were two cows on board a fresh supply could be had for patients who were unable to take the sterilised milk.

### PERSONAL DETAILS.

Finally, I asked the sisters whether they contemplated returning to South Africa?

"Sister Fisher and I both hope to return to the Cape in about a month," Sister Laurence said. "My place as Superintendent of the Princess Christian Hospital was filled *pro tem.* by the appointment of Sister Fraser. I was superintendent from March till December 3rd, when I was offered the trip home, with the P.M.O., Major Mathias, D.S.O. With him came also, besides us two sisters, two sergeants and six orderlies of the R.A.M.C.

"For many things," said Sister Balfour, "I should like to return; but I cannot possibly do so, and I also feel that I should not stand a good chance of promotion at home if I continued military nursing."

"Do you consider that the war has been a useful experience? You do not think it likely to unfit a nurse for her ordinary work?"

"I certainly think the war has been a useful experience, but I do think it is apt to make one feel unsettled as regards hospital life and civilian nursing. I have enjoyed it all very much, but I am not going out again, though I have been asked to do so."

"Were you long in South Africa?"

"I went out on March 24th, 1900, and was sent up to Kimberley with nineteen other nursing sisters the first week in April. In the middle of September I went down to No. 1 General Hospital, Wynberg, and was there till December 10th, when I embarked on the ss. *Canada* for duty on the voyage."

### The Royal National Pension Fund for Nurses.

OUR readers who are interested in the development of the Royal National Pension Fund for Nurses will peruse the following quotation from the *Times* of January 5th with gratification:—"The latest figures of this Fund, of which the Prince of Wales is President, and in which Her Royal Highness takes a warm interest, show that it is now the greatest women workers' saving fund in the world. Its invested funds exceed £550,000, over £9,800 have been distributed in sick pay, and more than £90,000 returned to nurses who have been obliged to give up their policies from one cause or another. During last year 700 new policies were issued, £74,000 were received in premiums, £4,400 were paid in pensions, and £1,450 distributed as sick pay. Since the Pension Fund was only established in 1888, these figures show what remarkable progress it has made."

### Mr. Burdett-Coutts, M.P.,

AND

### "The Hospital" Nursing Mirror.

In his book just published, "The Sick and Wounded in South Africa," Mr. Burdett-Coutts, M.P., refers to the interview with him which appeared in our columns. He says:—"Earlier in the history of these events I had given the following interview to a journal which circulates largely amongst the nursing community. I had maintained the same abstention from 'interviews' as from popular audiences, but the subject of female nursing in military hospitals was one of sufficient importance to tempt me to make an exception in its favour."

After quoting the whole of our report Mr. Burdett-Coutts adds a few words about the sentimental difficulty as follows:—"What I have called the 'sentimental difficulty' is a real one under the peculiar conditions of a campaign. It is not insuperable, but it requires the careful consideration of wise and practical women, so many of whom, both English and Colonial, have rendered noble service to the cause of humanity in this war. If some of these were to meet in conference with some of the superintendents and elder nursing sisters who have had the experience of the campaign, a satisfactory scheme for the future could be easily drawn up. It is essentially a matter for women to settle amongst themselves; but if I might venture to make a suggestion it would be on the following lines:—

"1. Careful selection.

"2. An experienced and acceptable matron or superintendent for each contingent of nurses, with full power of discipline and dismissal.

"3. The continued solidarity, as far as possible, of every such contingent under its own superintendent in any particular hospital to which it may be attached. It need hardly be added that when once the pressure arose, none of these safeguards attended the provision of nurses in this campaign."

### Where to Go.

The Dowdeswell Galleries.—A new sacred picture by William Strutt is being shown at these galleries, and also a collection of water-colour drawings by C. J. Collings.

The New Gallery.—The present season exhibition at the New Gallery is devoted to the works of Sir W. B. Richmond. A new departure is announced in connection with the gallery. It now combines the attributes of a club with those of a picture gallery. Season-ticket holders will have the privilege of introducing friends. Facilities for correspondence, magazines, papers, and refreshments are provided.

### Minor Appointments.

Jessop Hospital for Women.—Miss Elizabeth Hickman has been appointed sister. She was trained at the Royal Infirmary, Derby; the Wolverhampton and District Hospital for Women, and the City of London Lying-in Hospital. Subsequently, she was charge nurse at the Wolverhampton and District Hospital for Women, but for some time lately she has been engaged in private nursing.

Swansea Union Workhouse Hospital.—Miss Charlotte Calver has been appointed nurse for the female block. She was trained at the Royal Infirmary, Glasgow, and has since been engaged in district nursing.

The Union Infirmary, Newton Abbot.—Miss Ellen Kavanagh has been appointed assistant nurse. She was trained by the Meath Workhouse Nursing Association.

### For Reading to the Sick.

"Do not think it wasted time to submit yourself to any influence which may bring upon you any noble feeling."—*T. Ruskin.*

Meanwhile with every son and saint of Thine
　　Along the glorious line,
Sitting by turns beneath Thy sacred feet
　　We'll hold communion sweet,
Know them by look and voice, and thank them all
　　For helping us in thrall,
For words of hope, and bright examples given
To shew through moonless skies that there is light in heaven.—*J. Keeble.*

Still may Thy sweet mercy spread
A shady arm above my head,
About my paths; so shall I find
The fair centre of my mind
Thy temple, and those lovely walls
Bright ever with a beam that falls
Fresh from the pure glance of Thine eye,
Lighting to eternity.—*R. Crashaw.*

If we cannot live at once and alone with Him, we may at least live with those who have lived with Him; and find, in our admiring love for their purity, their truth, their goodness, an intercession with His pity on our behalf. To study the lives, to meditate the sorrows, to commune with the thoughts, of the great and holy men and women of this rich world, is a sacred discipline, which deserves at least to rank as the forecourt of the temple of true worship, and may train the tastes, ere we pass the very gate of heaven. . . . We forfeit the chief source of dignity and sweetness in life, next to the direct communion with God, if we do not seek converse with the greater minds that have left their vestiges on the world.—*J. Martineau.*

Make yourselves nests of pleasant thoughts. None of us yet know, for none of us have been taught in early youth, what fairy palaces we may build of beautiful thought—proof against all adversity. Bright fancies, satisfied memories, noble histories, faithful sayings, treasure-houses of precious and restful thoughts, which care cannot disturb, nor pain make gloomy, nor poverty take away from us—houses built without hands, for our souls to live in.—*J. Ruskin.*

For nowhere either with more quiet or more freedom from trouble does a man retire than into his own soul; particularly when he has within him such thoughts, that by looking into them he is immediately in perfect tranquility. And I affirm that tranquility is nothing else than the good ordering of the mind.—*Marcus Antoninus.*

Ah, holy midnight of the soul,
　When stars alone are high;
When winds are dead, or at their goal,
　And sea-waves only sigh!
Ambition faints from out the will;
　Asleep sad Longing lies;
All hope of good, all fear of ill,
　All need of Action dies;—
Because God is! and claims the Life
　He kindled in thy brain;
And Thou in Him, rapt far from strife,
　Diest and liv'st again.—*MacDonald.*

### To Nurses.

**We invite contributions from any of our readers, and shall be glad to pay for "Notes on News from the Nursing World," or for articles describing nursing experiences, or dealing with any nursing question from an original point of view. The minimum payment for contributions is 5s., but we welcome interesting contributions of a column, or a page, in length. It may be added that notices of entertainments, presentations, and deaths are not paid for, but, of course, we are always glad to receive them. All rejected manuscripts are returned in due course, and all payments for manuscripts used are made as early as possible at the beginning of each quarter.**

### The Ordering of Hypodermic Injections.

ONE of the duties which a nurse may often have to perform is the giving of hypodermic injections of morphine or other drugs, and in doing this, of course, she must take the greatest pains to carry out accurately the instructions she receives, and to give the exact dose that is ordered. On the other hand, a duty lies upon the practitioner to give these instructions in such a form that, without gross carelessness, no accident can happen in carrying them out. A nurse has forwarded to us the following question, which seems to involve points of too great importance to be answered in our "Queries" column. She says:—"I was trained at a hospital where hypodermic injections were given in minim doses. The practice of giving them in grain doses puzzles me. Will you kindly tell me where I shall find what the difference is, explained?—*Puzzled*."

Now, in answering this question we would first point out certain elementary facts. The minim is a measure of bulk and is used exclusively for the measurement of liquids, while the grain is a measure of weight. Naturally, therefore, a practitioner ordering an injection, which is a fluid, would order it in minims. But owing to the fact that solutions of various strengths have from time to time been employed, and, indeed, are still employed, some practitioners prefer to prescribe the weight of the actual drug which they desire to be injected, leaving it to the dispenser to arrange about the strength of the solution and the quantity of it which will require to be given to get the dose which has been ordered.

Of late years, again, certain druggists have introduced "tablets" or "tabloids" or "lamels," each containing a given quantity of the drug, measured, of course, in grains. Sometimes these are first dissolved in a small quantity of water, which is then drawn into the syringe; sometimes the tablet is dropped into the syringe and the water then added, but in either case the dose in each tablet is marked in parts of a grain, and when such preparations are to be used the dose should be prescribed also in parts of a grain. No physician ought to entrust the giving of a hypodermic injection to a nurse or, indeed, to anyone, unless he takes care that the dose is ordered in weights or measures applicable to the form in which the drug is dispensed—in minims if in the form of a solution, in fractions of a grain if dry. If, however, it should happen that a nurse who has been told to give so many minims finds herself provided only with tablets, or if, when told to give a fraction of a grain she finds herself provided only with a solution, then she must ascertain the strength of the solution. In our opinion such careless prescribing is utterly wrong and throws upon a nurse a responsibility which ought never to be placed upon her, and which she will do well to decline; but for the information of "Puzzled" we may say that, according to the British Pharmacopœia, each 110 minims of hypodermic injection contains the following quantities of its active drug:—In the case of apomorphine, 1 grain; in the case of cocaine, 10 grains; in the case of ergot, 33½ grains of the extract; and in the case of morphine, 5 grains.

From this it will be seen that such quantities as the third, or fourth, or fifth, or sixth of a grain cannot be got in whole minims. At first sight it may seem an extraordinary thing that any sensible body of men should ever have arranged for the solutions to be of such strengths that there should be no simple relationship between them and the amount of the drugs which they contain. As a fact, however, the relationship is very simple, namely, in the case of the morphine injection 5 per cent., or one in twenty.

The root of the difficulty is that there is no simple correspondence between the weights and the measures used in England. A fluid ounce of water weighs an ounce, but while there are 480 minims in that fluid ounce there are only 437·5 grains in it by weight. Thus a grain and a minim have no relation to each other, and from this comes all the difficulty. From the practical point of view no one can doubt that the old plan, in which there was a distinct and simple numerical relationship between the grains of the drug and the minims in the solution, was infinitely the best. Take, for example, the very common strength of 1 grain in 12 minims; with this every ordinary fraction of a grain except the fifth and the seventh could be given in whole minims—and nobody wants to give fifths and sevenths. At present everything is in an absolute muddle, and as far as we know the only way in which it is possible to give hypodermics with exactitude is to make use of the tablets which dissolve in the syringe; and in regard to them it must be remembered by the nurse that the tubes containing these tablets are very small and the figures on them very minute, so that the greatest care must be taken to make sure of using the tablets of the proper dose. In any case it is clear that it is next to impossible to transpose off-hand from solids to fluids or *vice versâ*, and if at any hospital the practice of leaving this to the nurses has been allowed to grow up, the matter ought to be at once brought before the medical staff by the matron.

It is a mere matter of laziness on the part of the doctor. If he wants his patient to have a quarter of a grain of morphine, for example, 5 minims is too little, while 6 minims is too much. It is for the doctor, not for the nurse, to decide whether the dose shall be this little bit over or this little bit under the quarter of a grain. But there is another and much more serious aspect of the question. In the year 1898 the new Pharmacopœia entirely altered the strengths of the injections, making that of morphine only about half the strength of the solution previously in use. But there are still many medical men who prefer the stronger forms. Although the official form contains only 1 grain of tartrate of morphine in 22 minims, Martindale's "Extra Pharmacopœia"—a book far more used by prescribers, we should think, than the official book—gives the strength of the injection of acetate of morphine as 1 grain in 6 minims. When, then, a nurse is told to give a quarter of a grain, say, of morphine and is given for the purpose a bottle containing a solution, unless the strength of this solution is plainly printed on the bottle she may easily be led into making a disastrous mistake; while even if it is so printed she will be in difficulties unless she is prepared to calculate the dose according to the strength given, and is still further prepared to take the responsibility of deciding whether to give more than the dose or less than the dose ordered so as to bring it into even minims. Unless, indeed, she feels equal to the tickle task of splitting a minim. The whole thing is utterly and entirely wrong. A practitioner may quite properly leave the giving of a hypodermic injection to a nurse whom he knows and can trust. But he cannot be held free from blame unless he takes the trouble to tell her exactly how much to give of the particular preparation with which she is supplied.

### "The Hospital" Convalescent Fund.

THE Hon. Secretary acknowledges with thanks the receipt of 1s. from Mrs. Frances Swinton.

### Echoes from the Outside World.

AN OPEN LETTER TO A HOSPITAL NURSE.

Now that the New Year has turned, the arrangements for the Queen's annual spring expedition are being pushed on apace. It seems as if Cimiez will, after all, be the favoured spot, much to the disappointment of the Bordighera folks, who had hoped that their little village would have been her honoured resting place this first spring of the new century. Nice, too, has made a representation through its mayor and municipality how delighted they would be if the Queen of England would visit them in March; but the mere fact of the popularity of this well-known winter resort is sufficient to influence Her Majesty in her decision, as it has influenced the Czar of Russia. People are so apt to forget that when Royal folks go for a holiday it is not only change of air they want, but also change of society and rest and quiet. All these it is impossible to obtain if the place is full of Royal visitors and members of noble houses, who must be called upon and entertained, and with whom it is quite impossible to avoid constant intercourse more or less tiring to invalids and those no longer young. Consequently, our own Sovereign chooses some less frequented spot for her stay than the Riviera proper, and the monarch of all the Russias is likely to go to Corsica to get up his strength after his recent severe illness. As to Ireland, the Queen has again shown her kindly solicitude for all her subjects, and her wish to please them if she can possibly do so. Finding that the warm-hearted and enthusiastic Irish people were bitterly disappointed that she was not coming again this year, Her Majesty impressed upon the Prince of Wales the desirability of his projected visit to Ireland taking place about the same time as her own stay last year, with the result that he and the Princess wil go to Dublin, as guests of Lord and Lady Cadogan, during April. This will go far towards making up for the absence of the Queen herself, and a most cordial welcome will certainly be accorded to the Heir Apparent and his wife.

---

It is distinctly a matter for congratulation that Earl Roberts chose the first week of the new year to arrive in England rather than the second, for though the familiar London fog did something towards spoiling the procession from the station, it was much more of a success than would have been possible in the snow and ice which unexpectedly put in an appearance later on. Jack Frost seems to have been strangely impartial in his attentions, Rome and Venice, Paris and Vienna, Marseilles and London, all experiencing bitter cold. At Rome the snow mantle which covered the city occasioned exceeding glee, the citizens not having seen such a pretty sight for seven years, and everywhere skaters and tobogganers became of course temporarily happy; but the traffic in many places has been stopped, and a large number of deaths from exposure are recorded. Many men endeavour to keep themselves warm by extra smoking, though it is doubtful if the kindling of a little fire outside the mouth causes a corresponding glow in the feet. Anyhow, having arrived at years of discretion they may do as they choose, without the reprimand so opportunely given by Earl Roberts to a small boy at Swindon Station, on Saturday, who stood near him smoking a cigarette. The Commander-in-Chief told him that "it was very rude of a boy of his age to smoke." If I were much associated with lads, I should try and found a "Bobs' Anti-smoking League" upon this incident, and I think, so much is he an idol with all schoolboys at present, that it would be successful.

---

The dawn of a new century seems to have suggested to the authorities at Burlington House to collect together some of the works of art of the past fifty years, and the hundred odd oil paintings, and two hundred water colour and black and white drawings now to be seen in Piccadilly have all been painted by artists who have died since 1850. The Queen has lent the portrait of herself, painted by Sir Francis Grant, which depicts her with the Prince of Wales as a tiny babe upon her knee, whilst the Princess Royal is feeding a couple of dogs with biscuits, and the Prince of Wales contributes a fairly well-known work from the brush of Sir Edwin Landseer, called "The Connoisseurs" representing the artist sketching while a couple of dogs look over his shoulder and criticise his handiwork. "The Stag at Bay" is an old friend, and most of us are so familiar with it in the engraving that it is pleasant to come across the original drawing. Another picture by Sir Edwin is the "Midsummer Night's Dream" showing Titania and Bottom with their attendant fairies, and it is a most strange and beautiful piece of colouring. There are also some well-known Turners—the "Minotaur," an awe-inspiring realistic shipwreck, and "Venice," full of the sunshine and atmosphere in painting which this artist was an adept. In Millais' diploma work, "Souvenir of Velasquez," the pretty little girl in her bright Spanish costume causes one to realise that the work which was painted to justify the artist's admission into the Academy did so in very truth, and was a fitting prelude to "The Gambler's Wife," "Winter Fuel" and "Merry," all of which are just now housed under the same roof. Of Lord Leighton's work there is not much, only "The Slinger" and a "Study of a Girl's Head." The single Rossetti, "The Vision of Fiammetta" makes one long for another, so magnificent is the feast of colour. One of the most beautiful sea pieces in the exhibition is Hamilton Macallum's "Water Frolic," boys bathing in the sea, which is instinct with life and happiness, and throws into strong contrast Frank Holls' masterly canvas redolent of grief, "The Lord gave, and the Lord hath taken away." The former has improved wonderfully by age, and the story which it tells, so simply, so sorrowfully, and yet so naturally, seems to come again with fresh force to the beholder after the lapse of years. Unfortunately, the same cannot be said of either of Albert Moore's works, "The Quartette" and "The Summer Night," or of Cecil Lawson's "The Storm." In the latter the colour does not appear to have improved; it seems to have grown dark and heavy, whilst the daintiness and freshness of the former artist's productions appear to have lessened. This, however, may be a case of recollection having beautified a work beyond its actual merit. But all nurses who love pictures should not fail to go to the Royal Academy and judge for themselves, for the collection has much to please and much to instruct, and it is well sometimes to refresh one's memory as to what has been done in the past.

---

A new avenue has been opened to Russian women desirous of earning their own livelihood. It appears that the authorities of the Riazan-Ural Railway have experienced a great deal of difficulty in finding in that part of Russia educated and reliable men to fill official positions, with the result that the chief of the board has appealed to the Minister of Communications for leave to employ women who have been passed by the Railway School at Saratof as station-masters, sub-station-masters, luggage inspectors, and telegraph superintendents. The necessary permission has been granted, and it will be interesting to learn in a few months' time how the experiment succeeds. It should do admirably, for none of the work requires great physical strain or endurance, and care, thoroughness, and business habits are the most requisite qualities; much the same as in many other positions which women, at least in England, have already filled with credit. As ticket clerks they should do excellently, and the only difficulty they are likely to experience in the other rôles specified will probably arise if they have a good many males of a rough class under them. These men might resent their superior position, and stir up many troubles. I do not, however, know enough about the duties of a station-master to be sure that this is more than an imaginary drawback to the new departure. As to England, I should not be surprised if the example set by Russia were copied by some of our English railway companies, more especially if the present disposition on the part of the men to strike continues. There is already one female station-master who discharges her duties admirably, and there is no reason why others should not be created.

### The Work and Diet of Nurses and Probationers in Hospitals.

A LETTER BY MISS LOUISA TWINING.

Fifteen years ago I wrote a paper on this subject which was read at a meeting of the Hospitals Association, at which many matrons of hospitals and workhouse infirmaries were present and took part in the subsequent discussion. I believe that since that time some considerable progress and improvement has been made, but that much still remains to be done will not, I think, be disputed by those who are acquainted with the present state of things, and thus I ask to be allowed to make a few remarks, in all friendliness, and with a knowledge of facts.

THE PROGRESS IN THE ART OF NURSING.

I have watched the progress that has been made in the art of nursing ever since its dawn nearly fifty years ago, when the Gamps of the nursing world, and the pauper so-called "helps" began to disappear and give place to others more worthy of the name, a former state of things which can hardly be imagined at the present day. About this time, viz., nearly fifty years ago, the cause was helped by one who is nearly forgotten by the present generation, Mrs. Jameson, who gave, and subsequently published, two important lectures on women's work and the "Communion of Labour," with an introductory letter addressed to Lord John Russell, as preface. I venture to give an extract from this remarkable expression of opinion, given so long ago, as it embodies in few words much that I wish to say. She had been speaking of recent experiments in prison discipline, and adds, "Englishwomen are of opinion that the principle of an interfusion of female, or what might be called, *maternal*, management, is even more obviously applicable to other institutions. For instance, there are two hospitals in London for the treatment of women and female diseases which are governed by men only; and, what is a still more curious anomaly, we have a hospital for sick children, in which the constituted authorities consist of 26 men (lords and gentlemen) and one woman in a subservient position. . . . I have myself seen gentlemen walking about and examining into all the kind and motherly arrangements for infant accommodation. There is something strangely absurd in all this." She then goes on to comment on the fact of a hundred benevolent gentlemen dining at the Freemasons' Tavern for the benefit of the poor sick babies, while ladies sit up in a sort of dark cage and look on, and ends by asking "how long the capacities and privileges of women are to be lowered in the estimation of the community by shutting them out of what is surely within the 'woman's proper sphere?'" At the time this was written, it might not have been easy to find women competent for these duties, but this can hardly be urged as a reason at the present day. Mrs. Jameson ends her eloquent letter by saying that Lord John Russell, and many others, have openly expressed the opinion that "it is to women we must look for the regeneration of society."

WOMEN ON THE BOARD OF MANAGEMENT.

The words I have put at the head of this communication will explain the truth that I feel so deeply and wish to enforce, that if our hospitals and similar institutions are to be managed rightly and justly, as all desire they should be, we must have introduced into all, what is acknowledged by some to be of great benefit and advantage, the presence of women on the boards or committees of management. In a recent long correspondence in *The Times* concerning hospital matters, several secretaries gave their testimony as to the value of women's advice and counsel in the management. If it is argued, as it has been during past years, that such an arrangement would cause "embarrassment," confusion, or something worse, I ask to give the testimony, universally accorded during the last twenty-five years, to the results of the admission of women as members of boards of guardians throughout the country. They number now no less than one thousand, and will every year be added to; their work, mainly now amongst the sick, the aged and children, is of the same nature as that of boards of management in hospitals, and personally, I can testify as to what has been done in the way of reform and economy during all these years. And why should this be doubted? Have not women been the "housekeepers" of the world through all time, and if so, why should they be so on a small scale when they are even more urgently required in the larger and public institutions where precisely the same qualities are required?

TWENTY YEARS' EXPERIENCE.

In the twenty years' experience of our "Workhouse Nursing Association" I have seen the necessity for having women, not only on Boards of Guardians, but also as inspectors of workhouses, a matter so strangely delayed, after the first experiment was made, with such absolute success, twenty-five years ago. How can even the best and most qualified men look into the state of things in these wards of the sick, and women and children, or how can the young women nurses speak to them with the freedom which is necessary, when they walk through the wards accompanied by the Master, and there is no one but the Matron for them to appeal to? Surely it is a matter of common sense which I urge, as to the absolute importance and necessity of adding a woman inspector (called a "sub-inspector" if so desired) to supplement the work of men, who if asked to perform such "domestic" duties in their own homes would scorn the task and refuse to perform it. It may seem as if I had left my subject, but I think the connection of the two questions will be acknowledged.

ALLEGED GRIEVANCES OF PROBATIONERS.

The treatment of nurses and probationers will surely be recognised as calling for the judgment and consideration of persons of their own sex, and till this power and privilege is granted we shall continue to have the grievances which now exist. I have cases now before me, known and well authenticated, of overwork and underfeeding in hospitals, especially of the young women probationers, the same as for some years past. The complaints come not from selfish or self-indulgent girls brought up in luxurious homes, but from earnest workers desirous of doing their duty in their future career, and in some instances used to active work in home management, but who have been obliged to give their training up after a short trial. Yet some of these could not live or do their work without going out for food, or receiving weekly hampers from home. In this connection I may add the serious danger of having recourse to stimulants to supplement the deficiency. Then as to the work—can it not be taught gradually, and the hard trial of standing for hours together be learnt by degrees, rather than all at once, resulting, as I know at the present time, in compulsory retirement after a few weeks' trial? Cannot half an hour be always allowed for dinner instead of 20 minutes, the time fixed in some instances? When so many ailments are now traced to defective digestion, is not the cause too often to be found in rapid consumption of food and want of due mastication? and, if so, what cause can be more likely than this? It was, I believe, said (when an inquiry was once made in the *Times*) that it was part of the duties of the Prince of Wales' Hospital Committee to investigate the care and condition and food of the nurses; but even if this is the case, does not the whole matter turn upon the question I have been putting throughout this paper—are men the best, or even competent, judges of matters which solely concern women and their welfare? I have had personal experience on two Boards of Guardians, in London and the country, and I venture to say they are not. I will give one instance from my own experience, though I do not wish to speak of myself: I was the first and only person who visited the nurses' dining-room, and discovered the errors of their diet, which was thus revised to their greatly improved health and comfort, and to the reduction of expenditure as well. Thus will it always be when the "communion of labour between men and women" is recognised and carried out in all the institutions of "charitable England"—a right and just principle, which I hope still to live to see become an accomplished fact, instead of, as at present, a disputed privilege.

### Guy's Hospital.

RESIGNATION OF MISS YOUNG, THE MATRON.

We regret to learn that on December 8th last Miss Young resigned her position as matron of this hospital. Her resignation was accepted by the Treasurer on December 22nd, and by the Court of Governors on December 27th, 1900, when the Court resolved: "That, in accepting with regret Miss Esther Young's resignation, the Governors place on record their cordial appreciation of her valuable services as Assistant Matron, and their recognition of the energy, industry, and careful attention to details of administration exhibited by her as Matron of the Hospital. Whilst successful in maintaining a high standard of discipline and conduct amongst those under her charge, Miss Young continued to enjoy the esteem and confidence of the nursing staff by whom the Governors have reason to know that her resignation is generally regretted."

A nursing correspondent writes to us as follows:—

"We are extremely sorry to hear that Miss Esther Young, Matron of Guy's Hospital, has resigned her appointment, and that her resignation has been accepted by the treasurer and house committee. Miss Young will be an irreparable loss to the nursing staff of the hospital with whom she was held in the highest esteem, and whose welfare she had always peculiarly at heart. During the time Miss Young has been connected with Guy's Hospital as assistant matron and matron, many improvements have been made for the nurses' comfort, which are owing to the great capabilities of organising and administration which she possesses in such a very high degree. The discipline of the hospital has been maintained at a very high standard, and it will be long before anyone will be found who will obtain the love and respect of the nursing staff as Miss Young has done."

### Everybody's Opinion.

[Correspondence on all subjects is invited, but we cannot in any way be responsible for the opinions expressed by our correspondents. No communication can be entertained if the name and address of the correspondent is not given, as a guarantee of good faith but not necessarily for publication, or unless one side of the paper only is written on.]

RULES IN PRIVATE NURSING.

"L." writes: Having entered on my nursing life within a month of sixteen years ago, may I be permitted to give my opinion and experience on the question as to whether a nurse should or should not sleep in a convalescent male patients' room. If a nurse is faithful and true to her duties, it will invariably prove to be one of the greatest comforts to a convalescing patient to have her within speaking distance during the night, and in many cases her very nearness, even though asleep, would induce a confident restfulness which no handbell, however quickly answered, could supply. Gratitude for services well and faithfully performed would, in her patients' mind, shield her from the slightest breath of questioning conventionality in the matter—he would only be too thankful not to have her frightened off, and leave him, possibly, to pass many weary, sleepless, hours alone, and he would be the first to endorse "Honi soit qui mal y pense." But should unhappily a nurse be sent to a male patient who during the day reminded her of her womanhood by word or look, her natural, or acquired, necessary tact should suggest to her ways and means of making him realise her inviolable insulation with regard to all ideas of sex; and with careful treatment, by the time he was convalescent she need fear nothing. I have heard so-called nurses indignantly protest that nothing would ever induce them to sleep in a male patient's room, and invariably my experience of them has made me feel it to be rather a pity that such women belonged to a profession which they were eminently unsuited to adorn. "Thistle's" ideas of giving four-hourly medicines are, I hope, not likely to be followed by many nurses. Say, for instance, that the medicine due to be given at two o'clock was not administered because the patient did not awaken until after half-past. In that case, "Thistle" says she should not give the dose before six o'clock. But supposing the patient was fast asleep again at six o'clock, and did not awaken before half-past, what would she do then? And how much benefit would the poor patient under her care derive from the medicine if he happened to sleep at inconvenient times?

SHADOWS OF THE WAR.

Mrs. Bagot, Levens Hall, Milnthorpe, writes: A most gratifying review of my book in your paper has been brought to my notice. It contains, however, one slight inaccuracy which I am anxious to correct with your kind permission. In alluding to the arrival of patients at a hospital which had only just been established, I have described the disappointment of the Sisters on receiving no stretcher cases, which was, after all, very natural under the circumstances. I should like to say that the incident is in no way connected with our hospital, as your reviewer implies.

BEEF TEA.

"Ancient Briton" writes: I learned to make beef tea in the following way. Take a pound of steak for typhoids, shin of beef for surgical cases, remove all fat, shred it and put into a stone jar in the oven with a pint of water—no salt. Let the beef simmer for an hour, then pour off all that will come clearly. Next put on enough water to cover the meat and stew till cooked. Steak will finish in one hour, shin requires two or three, and filling up the jar with cold water. "Never use salt except in the patient's cup or basin."

THE DEGRADATION OF NURSING.

An "Ex-Nurse" writes: There is a subject called "Home Nursing" taught by unqualified folk in County Council and School Board classes all over the country, and written about by ignorant persons in the lay press and in silly booklets. And there is no body of trained nurses to whom to appeal when the evil becomes positively indecent and dangerous. In America an appeal to the "Associated Alumnæ of Trained Nurses" would secure an immediate redress. Here we have two or three rival societies, such as the British Nurses and the Matron's Council, with two or three lady superintendents on each, but with the names of women such as Miss Luckes and Miss Gordon absent from all. And so when unqualified men are allowed by the London School Board to teach home nursing to girls and women I have nobody to whom I care to appeal (save the public through your pages) to help me express the indignation I feel at the degradation of my profession and the insult to my sex.

FOR READINGS TO THE SICK.

"Nurse P" writes: As I read this week's Hospital I am wondering how many nurses it reaches this New Year who are "called apart." I have often cheered my patients by your "Readings for the Sick." Most forcibly does the Divine message appeal to me this week, for I myself am numbered with the sick. I too, perforce, am among those who must spend this season in bed. Hard as suffering is to bear one cannot but feel that it is good for nurses to sometimes suffer. We rush on in hospital; operation follows operation. We have no time to enter into the individuality of our patients. We are anxious that each shall be a "good case," with speedy convalesence, and there, too often, our interest ends. Now I myself have had, what is professionally termed, "only a minor operation," yet please God when I recover and resume duty it will I hope be as a better and more sympathetic nurse. I am sadly afraid that we nurses, rushing on year in and year out get to regard pain and suffering too casually. We are so used to seeing others suffer, so used to seeing weary heads toss and turn. We do our duty, most of us do our best, and there it ends; we console ourselves that we have no time for more. Bodily pain renders all doubly sensitive; let us not be afraid of appearing too soft-hearted, and remember we read that our Saviour at the sight of suffering "groaned in His Spirit, and was troubled."

### Notes and Queries.

**The Editor is always willing to answer in this column, without any fee, all reasonable questions, as soon as possible.**

**But the following rules must be carefully observed :—**

**1. Every communication must be accompanied by the name and address of the writer.**

**2. The question must always bear upon nursing, directly or indirectly.**

**If an answer is required by letter a fee of half-a-crown must be enclosed with the note containing the inquiry.**

*"The History and Development of Trained Nursing."*

(161) Can you tell me if the series of articles on "The History and Development of Trained Nursing in the Nineteenth Century" can be had in book form ?—*B. N. S.*

No; but copies of the three issues containing the articles can be obtained from the publisher on receipt of 7½d. in postage stamps.

*Maternity Fees.*

(162) 1. I was engaged to nurse a lady in February, but owing to her premature confinement in December I was only able to be with her for two days. I charged her £1 11s. 6d., of which she paid me 15s. at the time, promising to send the balance. She has since refused to pay me anything more. Have I any redress? 2. I was engaged for November by a lady whose apparent pregnancy was caused by a tumour. She refused to believe this, and there being some doubt in the medical opinion, she held me to my engagement. When eventually my services were not required she refused to pay me anything either for fees, board and lodging, or compensation. The agreement was verbal, and I have only two letters on the subject from her. What can I do?

1. You can claim the balance through the County Court. 2. The absence of a written agreement makes it very difficult for you to substantiate your claims. Before putting the matter into the hands of a solicitor you would have to decide whether the money owing you would compensate you for the expense, loss of time, and worry inevitable in a law suit. In future have your scale of fees printed, and make sure that each intending patient has a copy at the time of engagement.

*Paying Probationer.*

(163) Is it possible for a paying probationer to enter into a London or provincial hospital for a short course of training ?—*A. S.*

Certainly. There are several hospitals which receive probationers for short terms of training upon payment.

*Sick Pay.*

(164) Is there any organisation which would help me in sickness, if I subscribed regularly to its funds in health? I am a trained nurse.—*Salisbury.*

Do you not belong to the Royal National Pension Fund for Nurses? There is a branch to ensure help in sickness to its members. Write to the Secretary, 28 Finsbury Pavement, E.C., for full information.

*Probationer.*

(165) I should be much obliged if you will kindly give me full particulars how to enter the London Hospital as nursing probationer without premium. —*C. V.*

The Matron, the London Hospital, Whitechapel Road, E., will furnish you with the desired information. The particulars are given in the "Nursing Profession: How and Where to Train."

*L.O.S.*

(166) Can you kindly give me the names of any institution in London where I might obtain the post of charge nurse, and at the same time see the cases and work for the L.O.S. certificate? I am a trained nurse.—*Enquirer.*

The Matron, Fulham Union Infirmary, St. Dunstan's Road, Hammersmith, W., might be able to help you.

*Matron's Maid.*

(167) Will you kindly tell me the duties and salary of a matron's maid, and f thirty is too old for that position ?—*C. C.*

The duties and salary would be similar to those of house-parlourmaid. The age would be very suitable.

*Canada.*

(168) Can you tell me anything about the training schools for nurses in Canada? I want to know the terms for training salaries, &c.—*Anglo-Canadian.*

You will find full particulars of the more important training schools of Canada in "Burdett's Official Nursing Directory," pp. 320-324. Salaries vary from *nil* to $108—that is about £21—for the first year.

*Advertising.*

(169) Will you kindly advise us as to the best way of making our medical, surgical, and convalescent home known? We wish to make it one especially for those recovering from infectious diseases, offering reduced terms to nurses, but fear that if we advertise in the usual way our neighbours may be alarmed.—*Kathleen H. A.*

Could you not work up a connection by sending your circular and references to medical men, nursing institutions, and societies working on sanitary and philanthropic objects? You could obtain addresses through the "Medical Directory," "Burdett's Official Nursing Directory," and the "Englishwoman's Year Book." You must be careful not to contravene the sanitary law, which is very strict in regard to the exposure or removal of patients who are still infectious.

*Aged Nurse*

(170) Can you tell me of a home for an old nurse (not an invalid). She could pay 5s. a week.—*Mrs. R.*

St. Edith's Hostel, Emscote, Warwick. Matron, Miss Butterworth. The Frithville Homes for reduced gentlewomen, 57 The Grove, Hammersmith, seem suitable, but see lists in the "Englishwoman's Year Book."

*Hypodermic Injections.*

(171) "Puzzled" may refer to page 200.

*Nurse-Child.*

(172) Could you kindly tell me of an institution or school where a child could be placed at moderate terms instead of being put out to nurse ?—*A. W.*

Dr. Barnardo's Homes, office, 18-26 Stepney Causeway, London, E. See the "Englishwoman's Year Book" for full lists of homes for this purpose.

*After Treatment.*

(173) Is it better to treat inflammation after confinement with hot or with cold compresses ?—*Maternity Nurse.*

This is a matter for the doctor.

*Croft Splint.*

(174) Will you please tell me what a "Croft splint" is ?—*A. A.*

A "Croft" is one of the most useful forms of plaster of Paris splint. It is made by taking four pieces of thick common flannel, the thicker the better, cut to the exact shape of the limb—two for the one side and two for the other. One piece of the flannel is applied to each side of the limb, and they are tacked together, front and back, quite loosely in such a manner that when this is done the limb is enclosed in flannel. Then a mixture of plaster of Paris in water is prepared, and the other two pieces of flannel are thoroughly soaked in it and made to take up as much as possible of the plaster. Just before the plaster begins to thicken these flannels are applied to the limb, one on each side, so that a plastered flannel just covers an unplastered flannel on each side. Then the whole limb is bandaged up, and it is a good plan to do this with a bandage which contains a little plaster, or to rub a little plaster over it—enough to make the turns keep their places, but not enough to prevent it being readily unwound. The great advantages of this splint are that it is very firm, and very light, and very easy to make—much easier than a Bavarian—and that when the limb shrinks within it, as almost always happens, it can easily be removed by unwinding the bandage without destroying or distorting the splint itself. This can then be re-applied and kept in position either by a bandage stiffened with a little plaster, or starch, or glue, whichever happens to be the handiest; or if frequent inspection is required, the splints may be held in position with strapping. If there is likely to be any swelling of the limb, a layer of cotton wool should be put on before applying the splint, and it is as well to remember that in cutting the flannel for a leg splint the stocking lying flat forms a good pattern. This admirable form of splint was invented by Mr. Croft, of St. Thomas's Hospital.

*Appointment Abroad.*

(175) Can you tell me the best means of obtaining an appointment as nurse-attendant at Cannes or Nice? Are there any agencies to which I could write ?—*Miss R.*

You might write to the Lady Superintendent of the Nice Nursing Institute, Villa Soleil, Avenue Thiers, Nice.

*Training.*

(176) Will you kindly tell me (1) if Kingston Union Infirmary, Surrey, is a recognised training school for nurses? (2) What advantages have nurses trained in a general hospital over those trained in a union infirmary? (3) Also what is meant by appointments being "subject to the provision of the Poor Law Officers' Superannuation Act?"—*Margaret J. A.*

1. No. 2. It depends entirely upon the individual institution. The training in some union infirmaries is first rate. 3. It means that compulsory deductions are made from the yearly salary in order to provide a pension in old age. Nurses are able to provide for the exigencies of age and sickness through the Royal National Pension Fund for Nurses, which would, of course, be in addition to what they would obtain under the Act.

*Electrolysis.*

(177) Can you tell me where I can get instruction in electrolysis? Are nurses ever trained at the electrical departments of the hospitals ?—*Sister M.*

Electrolysis appertains to medical and not to nursing instruction. There is no public institution which enables private persons to learn the art.

*Lecturer.*

(178) Will you tell me what training is required, and also to whom I should apply, for a post as lecturer on sick-nursing to the London County Council or the School Boards ?—*E. N.*

The County Council lecturers must be certificated teachers of their subjects. Apply Miss Ella Pycroft, 116 St. Martin's Lane, W.C. The Lecture Department of the Woman's Institute, 15 Grosvenor Crescent, S.W., is the association which can best help women aspiring to become lecturers.

*Africa.*

(179) Can you tell me to whom to apply for a Government appointment on the West Coast of Africa ?—*C. J. B.*

The Secretary, the Colonial Nursing Association, the Imperial Institute, S.W.

*Tallerman Treatment.*

(180) Can you kindly tell me if there is an institution in Yorkshire or in the provinces where a lady suffering from rheumatism could, by payment of a small fee, receive the Tallerman Treatment ?—*Lois.*

Mr. Tallerman would no doubt give information in regard to the places where his apparatus is in use.

**Standard Books of Reference.**

"The Nursing Profession: How and Where to Train." 2s. net; post free 2s. 4d.
"The Nurses' Dictionary of Medical Terms." 2s.
"Burdett's Series of Nursing Text-Books." 1s. each.
"A Handbook for Nurses." (Illustrated.) 5s.
"Nursing: Its Theory and Practice." New Edition. 3s. 6d.
"Helps in Sickness and to Health." Fifteenth Thousand.
"The Physiological Feeding of Infants." 1s.
"The Physiological Nursery Chart." 1s.; post free 1s. 3d.
"Hospital Expenditure: The Commissariat." 2s. 6d.
All these are published by THE SCIENTIFIC PRESS, Ltd., and may be obtained through any bookseller or direct from the publishers, 28 and 29 Southampton Street, London, W.C.

